# Continuous symmetry and chirality measures: approximate algorithms for large molecular structures

**DOI:** 10.1186/s13321-023-00777-x

**Published:** 2023-11-09

**Authors:** Gil Alon, Yuval Ben-Haim, Inbal Tuvi-Arad

**Affiliations:** 1 Department of Mathematics and Computer Science, The Open University of Israel, Raanana, Israel; 2 Department of Natural Sciences, The Open University of Israel, Raanana, Israel

**Keywords:** Symmetry, Chirality, Molecular descriptors, Supramolecular chemistry, Unit cells, Hungarian algorithm, Fibonacci lattice

## Abstract

Quantifying imperfect symmetry of molecules can help explore the sources, roles and extent of structural distortion. Based on the established methodology of continuous symmetry and chirality measures, we develop a set of three-dimensional molecular descriptors to estimate distortion of large structures. These three-dimensional geometrical descriptors quantify the gap between the desirable symmetry (or chirality) and the actual one. They are global parameters of the molecular geometry, intuitively defined, and have the ability to detect even minute structural changes of a given molecule across chemistry, including organic, inorganic, and biochemical systems. Application of these methods to large structures is challenging due to countless permutations that are involved in the symmetry operations and have to be accounted for. Our approach focuses on iteratively finding the approximate direction of the symmetry element in the three-dimensional space, and the relevant permutation. Major algorithmic improvements over previous versions are described, showing increased accuracy, reliability and structure preservation. The new algorithms are tested for three sets of molecular structures including pillar[5]arene complexes with Li^+^, C_100_ fullerenes, and large unit cells of metal organic frameworks. These developments complement our recent algorithms for calculating continuous symmetry and chirality measures for small molecules as well as protein homomers, and simplify the usage of the full set of measures for various research goals, in molecular modeling, QSAR and cheminformatics.

**Supplementary Information:**

The online version contains supplementary material available at 10.1186/s13321-023-00777-x.

## Introduction

Symmetry is an eye-catching phenomenon that expresses the beauty and mystery of nature. In chemistry, it is frequently perceived as a driving force that controls the shape of molecular structures, defines selection rules for the interaction of light and matter, and determines the terms and mechanisms of chemical reactions [1]. While perfect symmetry is conceptually appealing, numerous experimental and computational studies show that actual structures are only approximately symmetric [2–6]. Various reasons lead to distortion of otherwise symmetric molecules, including conformational flexibility, dynamics, chemical processes, physical conditions, crystallization conditions and the chemical environment. In many of these cases, describing the molecules at hand from the perspective of their deviation from the original symmetric geometry provides deeper understanding of the molecular systems. Such a treatment can highlight anomalous cases, and shed light on mechanisms of symmetry breaking. Mathematically, the description is based on the treatment of symmetry as a continuous quantitative parameter of the molecular structure, rather than a binary yes/no property. This approach was developed by Avnir and coworkers in the early 90's of the twentieth century, in the form of the continuous symmetry measure (CSM) [7–9], the continuous chirality measure (CCM) [10], and the continuous shape measure (CShM) [11]. In recent years we had considerably improved the algorithms that calculates the CSM and CCM for small-to-medium sized molecules [12] as well as for protein homomers [13]. Here we extend our methodology to large molecular systems which are beyond the reach of previous algorithms.

Given a distorted molecular structure and a symmetry point group *G*, the CSM algorithm searches for the nearest structure that belongs to *G* and maintains the same connectivity as the original molecule. The distance between the original structure and the nearest symmetric structure defines the symmetry measure. The continuous chirality measure follows by calculating the minimum CSM with respect to all the achiral point groups, *S*_n_. In the last three decades, this set of symmetry and chirality measures were extensively applied for describing various chemical phenomena in studies related to the crystal structure of inorganic compounds [3, 14, 15], reaction paths and reactivity [16–18], dynamics and temperature [19–21], protein structure and activity [22–24], quantitative structure–activity relationship (QSAR) [25, 26], and many more. Beyond measuring symmetry and chirality, the CSMs and CCM can be used to estimate structural elongation, planarity and conformational flexibility, for both symmetric and asymmetric structures [20, 24]. In addition, the method was applied to other fields such as image processing [27] and archeology [28, 29].

Finding the nearest symmetric structure is the main challenge of the CSM method, as it is unknown *a priory*, and may change even between conformers of the same molecule. This property turns CSMs into powerful global descriptors of the three-dimensional structure capable of distinguishing between various conformers of the same molecule. This ability stands at the heart of the concept of symmetry maps, an analysis tool for distorted structures [30, 31]. From a mathematical perspective, a structure that belongs to *G* is represented by a three dimensional vector (which represents the direction of the symmetry operation) and a permutation of the set of atoms (which represents the action of the symmetry operation on the molecule's atoms). As the number of atoms increases, the number of possible permutations increases as well, and calculating the CSMs becomes computationally intensive. To overcome this obstacle, we improved the algorithm for small-to medium-sized structures, by utilizing the connectivity map of the molecule to scan only structure-preserving permutations [12]. This improvement increased the accuracy and speed of the calculation, making the method applicable to much larger molecules compared to the original algorithm [8].

For very large molecular systems, with branched structures and complex connectivity maps, scanning all the permutations, even only the ones that preserve the connectivity map, becomes computationally intensive. For this purpose, Dryzun et al. [9] developed an approximate algorithm to calculate CSMs. Instead of searching for all possible permutations, their algorithm iteratively searches for an approximate direction of the symmetry element and its related permutation, until convergence is reached. While this approach is relatively fast, it may result with a permutation that breaks the connectivity map of the structure. To overcome this obstacle, we recently modified the method for protein homomers, utilizing the amino acids sequence to reduce the size of symmetry-equivalent groups of atoms, and force the code to preserve both the sequence and the chains structure. The Hungarian algorithm [32] was applied to efficiently solve the assignment problem and find the best permutation [13]. This approach is based on our prior knowledge of the protein sequence, and may become less effective when such information is absent, for example in the case of supramolecular structures and nanostructures. Finding the nearest symmetric structure in such cases without losing information on their connectivity maps requires a different methodology. In this study we present several algorithms for approximate calculation of symmetry and chirality measures that differ by their level of structure preservation and efficiency. Our set of algorithms provides a comprehensive toolkit for structural analysis that can be used to explore different sources of distortion, including conformational and topological distortion. In what follows we review the CSM methodology, present the details of the new algorithms, and use them to analyze three sets of molecules with various levels of approximate symmetry: pillar[5]arenes, C_100_ fullerenes and large unit cells of metal organic frameworks (MOFs).

## Methodology

### Review of the CSM method

Let us briefly review the fundamentals of the CSM methodology [7, 8, 12]. We consider a given molecule *A* of *N* atoms, that belongs to the symmetry point group *G*, where *G* is either *C*_n_ (n = 2, 3, 4, 5,…) or *S*_n_ (n = 1, 2, 4, 6,…). Recall that by definition, *S*_1_ = *C*_s_ and *S*_2_ = *C*_i_. Let ${{\mathbf{Q}}} = \left\{ {{{\mathbf{Q}}}_k :\,\,1 \le k \le N} \right\}$ be the set of coordinate vectors of the molecule's atoms, and let ${{\mathbf{Q}}}_0 = \frac{1}{N}\sum_{k = 1}^N {{{\mathbf{Q}}}_k }$ be its geometric center of mass. We are looking for a symmetry operation $T$, which generates a cyclic point group of type *G*. Note that *T* is a rotation (either proper or improper) by an angle of $360^{\circ} / n$. In both cases, $T$ is determined by a 3-dimensional direction vector, which we denote by $\nu_{sym}$. The continuous symmetry measure (CSM) is defined by:

1 $$S\left( G \right) = 100 \cdot M\left( G \right)/D$$

where

2 $$M(G) = \min \left[ {\sum_{k = 1}^N {\left| {{\mathbf{Q}}_k - {\mathbf{P}}_k} \right|^2 } } \right];\quad D = \sum_{k = 1}^N {\left| {{\mathbf{Q}}_k- {\mathbf{Q}}_0 } \right|^2 }$$

and the minimum is over all the symmetric (i.e. $T$-invariant) structures $\left\{ {{{\mathbf{P}}}_k :\,\,1 \le k \le N} \right\}$ and all possible direction vectors $\nu_{sym}$. Equivalently,

3 $$M(G) = \frac{1}{2n}\min \sum_{i = 1}^n {\sum_{k = 1}^N {\left| {T^i {{\mathbf{Q}}}_k - {{\mathbf{Q}}}_{\pi^i (k)} } \right|^2 } }$$

where $\pi$ is a permutation of the set of atoms $\left\{ {1,2,\ldots,N} \right\}$ which preserves the atom types and the molecule's connectivity map, and the cycles of $\pi$ are of size 1, 2, or *n*. Note that the value of 2 is only allowed when *G* = *S*_*n*_ or *G* = *C*_2_.

In our previous work [12, 13] we developed two algorithms for evaluating the CSM: The first one finds the exact value of *M*(*G*) in Eq. (3) by enumerating the structure preserving permutations, that is, permutations that satisfy the condition: *π(i)* ↔ *π( j)* if and only if *i* ↔ *j*, for all pairs of atoms (*i, j*). Here we denote by $i \leftrightarrow j$ the existence of a bond between atoms *i* and *j*. This algorithm, which we will call here the *exact algorithm*, shows excellent performance for small and medium sized molecules, and for larger molecules with a small number of structural symmetries [12]. For example, for fullerene, C_60_, all the atoms are in the same equivalence class. There are 2.73 × 10^43^ permutations that define a *C*_2_ operation, but only 32 of them preserve the structure of the molecule [12].

The second method focuses on protein homomers. Here the number of atoms does not allow scanning all structure preserving permutations. We therefore find an approximation for the CSM value by performing permutation-direction iterations (as explained below) [13]. A partial reduction of the number of permutations is achieved by exploiting the amino-acids sequence to define equivalence classes based on the types of the atom as well as its residue's designation and sequence number. Thus, a C_α_ of alanine with sequence number 3 on chain A can only be permuted with C_α_ of alanine with sequence number 3 on another chain, but not with C_α_ of different residues, or other alanine residues with different sequence numbers. The rest of the permutation is found using the Hungarian algorithm [32]. Further improvements are achieved by exploiting the protein polypeptide chain structure, making sure that the permutation does not break the chains, and carry each chain in its entirety to another chain: for protein homomers with more than two chains, the Hungarian method is used at the chain level as well, in order to find bijections between the different chains and piece them together to a complete permutation [13].

### Methods for general large molecules

As molecules become larger, the size of the equivalence classes increases, and the feasibility of a CSM calculation that performs an exhaustive search over all possible permutations reduces considerably. Unlike proteins, the atoms of large molecular structures do not have sequence identifiers that can help reduce the number of possible permutations. The challenge with such molecules is to find a good approximation to the CSM within a reasonable calculation time. We have developed several algorithms that successfully face this challenge, as described below.

### Permutation-direction iterations

Our algorithms are based on the process, first described by Dryzun et al. [9], of going back and forth between estimating the direction vector $\nu_{sym}$ and estimating the permutation $\pi$. An initial guess for a direction vector is based on the best line or plane that fits the geometric centers of all the equivalence groups of the molecule at hand, after outliers are removed. Two additional perpendicular direction vectors are taken into account in order to equally span the three-dimensional space [9].

Next, we perform an iterative process in which we update each time the permutation $\pi$ and the vector $\nu_{sym}$:

Given the current vector $\nu_{sym}$, the permutation is estimated by using a *greedy algorithm*. One first calculates the distance matrix $A = \left( {A_{ij} } \right)$, where

4 $$A_{ij} = \left| {TQ_i - Q_j } \right|^2 \quad {\mathrm{for}}\quad 1 \le i \le N,\quad 1 \le j \le N$$

and $T$ is the symmetry operation corresponding to the vector $\nu_{sym}$.

Let the smallest entry in the matrix $A$ be $A_{i_0 j_0 }$. The permutation value $\pi (i_0 )$ is set to $j_0$, and the row and column of this entry are greyed out. Then, the smallest entry from the remaining values in the matrix is chosen, to set another permutation value, and so on.

Given the permutation, the optimal vector for this permutation $\nu_{sym}$ is found by an exact analytical method [9, 12], which later serves to find a new permutation and so on, until convergence is achieved.

#### The Hungarian algorithm

A more sophisticated approach to finding the permutation, taken in our previous work [13] applies the Hungarian algorithm [32] to the matrix $A$. The algorithm finds a permutation $\pi$ for which the sum $\sum_{i = 1}^N {A_{i\pi (i)} }$ is minimal. This approach takes into account the interaction between different choices of the permutation values.

We should note that both approaches—the greedy algorithm and the Hungarian algorithm [32]—yield permutations that are not guaranteed to preserve the structure of the molecule. In practice, this depends on the symmetry level of the molecule. For molecules of high symmetry level, the approximate algorithms will tend to find the right permutation, and consequently, the bonding structure will be preserved, either fully or with a high preservation rate. For highly asymmetric molecules, this will not always hold, as permutations which do not preserve the bonding structure could attain lower values of the target function, thereby creating imprecisions in the CSM calculation. We elaborate more on this issue in the Results section.

#### The Fibonacci lattice algorithm

The original algorithm of permutation-vector iterations [9] starts with a direction vector which is an educated guess, hoping that through the iterations, the direction vector will converge to the optimal one. However, there is a risk that the iterative process will converge to a local minimum and not to the global one. In order to increase the reliability of the method, we devised the following strategy: A set of $k$ vectors on the unit sphere, $S$, is chosen such that the points are as equally spaced as possible on the sphere. A sequence of permutation-vector iterations is performed, starting at each of the vectors in $S$.

Finding a set of $M$ unit vectors which are equally spaced on the sphere is, in general, an unsolved mathematical problem. However, there is a well known way to produce $M$ unit vectors which are almost equally spaced. This set is called *The Fibonacci lattice* [33, 34]. It is defined as follows:

For any $$0 \le k \le M - 1$$,

5 $$\theta = \pi \left( {\sqrt {5} + 1} \right)k;\quad x = 1 - \frac{2k}{{M - 1}};\quad r = \sqrt {1 - x^2 }$$

6 $$u_k = (x,r\cos \theta ,r\sin \theta )$$

**Fig. 1:**
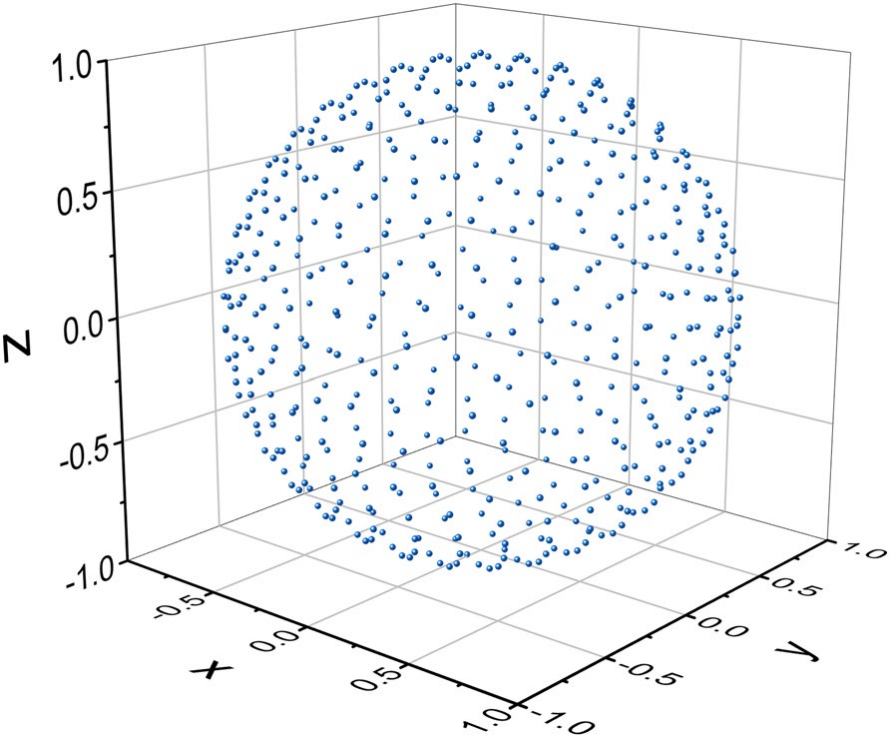
Fibonacci lattice of size 500

The set ${{\mathcal{F}}}_M = \left\{ {u_k |0 \le k \le M - 1} \right\}$ is called the Fibonacci lattice of size $M$. Figure 1 presents a Fibonacci lattice of size 500.^1^

We use all of the vectors in ${{\mathcal{F}}}_M$ as starting points for the permutation-direction iterations. The implementation of this algorithm can be sped up by parallel computation: The set ${{\mathcal{F}}}_M$ is divided into subsets, and each processor performs the iterations with the vectors in its assigned subset as initial vectors.

#### The approximate structure preserving algorithm

As discussed above, scanning only structure preserving permutations is considerably advantageous over the original CSM algorithm [7, 8], making it faster and more accurate [12]. However, for large molecules its running time can be too long for a feasible or efficient calculation. On the other hand, the approach of the approximate permutation-direction iterations algorithm is operative on any molecular size, but can often yield permutations that do not preserve the structure. In these cases, the nearest symmetric structure, which serves as the reference structure for the CSM calculation, may lose its chemical essence making the CSM result less informative. We now present a new algorithm, which attempts to bridge these two approaches and have the benefits of both. The algorithm performs permutation-direction iterations as in the approximate algorithm, but instead of using the Hungarian algorithm [32] for estimating the permutation, it searches for the best structure preserving permutation with respect to the distances matrix (4). As detailed below, this algorithm uses information from the distances matrix to prioritize the search for the permutation.

Recall that the exact (structure preserving) algorithm for small molecules [12] performs a recursive enumeration of the permutations in the following way: at each step, we define a partial assignment of permutation values; these assignments determine restrictions on the permutation values of the other atoms, according to the connectivity map of the molecule and the expected cycle structure of the permutation. An atom with a minimal number of permutation options is chosen, and for each option, this permutation value is assigned and the algorithm continues recursively.

In our new algorithm, which we call the *approximate structure-preserving algorithm*, in each permutation-vector iteration, the permutation is chosen as follows: We build the distances matrix (4), and perform a search for a structure preserving permutation as in the exact algorithm, but in the recursive step we prioritize assignment values $\pi (i) = j$ for which $A_{ij}$ is small: Given the options $j_1 ,\ldots,j_k$ for values of $\pi (i)$, we sort them according to the value $A_{ij}$ and perform the recursive calls from the minimum value and up. Whenever the algorithm reaches a full permutation $\pi$, the sum $S(\pi ) = \sum_{i = 1}^N {A_{i\pi (i)} }$ is calculated, and in the end, the permutation for which $S(\pi )$ is minimal is returned. As soon as the first permutation is obtained, the corresponding $S(\pi )$ is set as a threshold for the next partial permutations. For a partial permutation $\mu$, defined for a set $T$ of atoms, if the sum $\sum_{i \in T}^{\,} {A_{i\mu (i)} }$ is already larger than the minimal value of $S(\pi )$ so far obtained, there is no point in carrying on the search from $\mu$. By prioritizing assignment values with small corresponding entries in the distance matrix, we increase the likelihood of finding the best permutation fast. The algorithm contains a time limit, after which the search is terminated.

### Obtaining upper and lower bounds on the CSM

For large molecules, when the exact algorithm is unfeasible, it is advantageous to obtain upper and lower bounds for the CSM. Instead of bounding the expression $M(G)$ in (2), we bound the related expression

7 $$\widehat{M}(G) = \frac{1}{2}\min \sum_{k = 1}^N {\left| {T{{\mathbf{Q}}}_k - {{\mathbf{Q}}}_{\pi (k)} } \right|^2 }$$

In this expression the average in (2) over $i = 1,2,\ldots,n$ is replaced by the value for $i = 1$. Note that for $n \le 2$ (i.e. when *G* is *C*_s_ or *C*_i_), $\widehat{M}(G) = M(G)$, and for $n > 2$, $\widehat{M}(G)$ is a reasonable approximation of $M(G)$. Indeed, the approximate algorithms settle for minimization of $\widehat{M}(G)$ [9, 13].

Consider the approximate algorithm, paired with the Hungarian method [32] for finding the permutation, and the Fibonacci lattice method for the initial direction vectors, with a large number of initial points. We claim that this method produces a lower bound for $\widehat{M}(G)$ (which is a lower bound to the CSM when *G* is *C*_n_ or *S*_n_ with $n \le 2$). Indeed, if the Fibonacci lattice is dense enough, one of its vectors will be close enough to the optimal direction vector; and the permutation found by the Hungarian algorithm [32] for this vector, will satisfy $\frac{1}{2}\sum_{k = 1}^N {\left| {T{\mathbf{Q}}_k - {\mathbf{Q}}_{\pi (k)} } \right|^2 } \le \widehat{M}(G)$, as the Hungarian method finds the minimum over all permutations, not just the structure preserving ones.

Consider, on the other hand, the approximate structure-preserving algorithm. This algorithm calculates the minimum over some of the direction vectors $v_0$, and some of the structure preserving permutations $\pi$, of the expression $\frac{1}{2}\min \sum_{k = 1}^N {\left| {T{{\mathbf{Q}}}_k - {{\mathbf{Q}}}_{\pi (k)} } \right|^2 }$. Its value is, therefore, bigger or equal to $\widehat{M}(G)$.

## Results and discussion

In order to assess the efficiency and accuracy of the algorithms we tested them with three sets of molecules with various levels and sources of distortion. The first two sets were chosen so that both the exact and approximate CSM calculations can be performed, in order to compare the results and evaluate the performance of the various approximate algorithms. The first consisted of a set of highly flexible conformers of pillar[5]arene complexed with a Li^+^ ion in the gas phase. The second consisted of isomers of C_100_ fullerene. Different combinations of pentagons and hexagons that characterize the topology of each isomer, create diverse ellipsoidal-like structures with various symmetries. As a third set, we analyzed the crystallographic unit-cells of several MOFs, each with thousands of atoms, to test the applicability of the approximate algorithms on very large structures. For each set we calculated symmetry and chirality measures, the level of structure preservation, and the time of calculation. Details of each analysis are described next.

### Pillar[5]arene-Li^+^ complexes

**Fig. 2:**
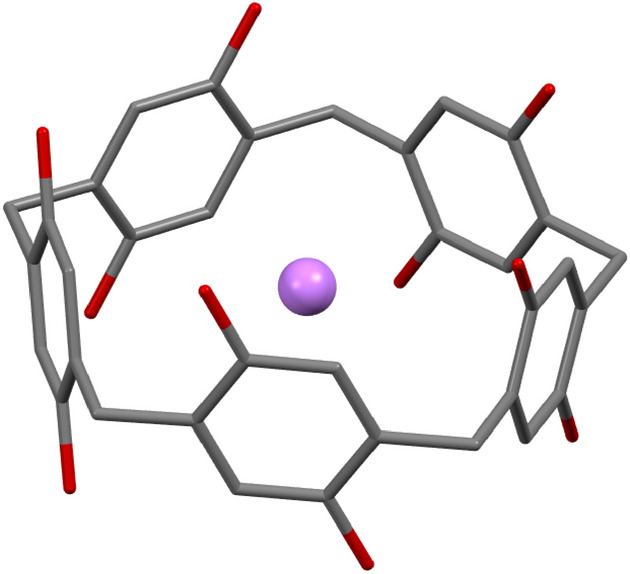
Pillar[5]arene with Li^+^ ion in a perfectly symmetric *D*_5_ conformation

Pillar [n]arene represents a class of supramolecular systems, originally synthesized by Ogoshi and coworkers [35, 36], with pillar-shaped architecture and double rim structure. Recent studies show that their superior host–guest abilities, planar chirality, and the ability to undergo self-assembly processes with highly symmetrical structures stem from their unique shape [37–39]. Pillar[5]arene, presented in Fig. 2, is the most common member of this family, for which five-fold rotational symmetry has been reported [36]. The high number of rotatable bonds gives rise to hundreds of conformers, which are generally close in energy. As will be shown below, the actual structure of these conformers can often be quite far from symmetry. In order to correctly describe the manifold of conformers of this molecular system, and distinguish between them, tools that provide a global description of the geometrical parameters are needed. Such description can contribute to better understanding of host–guest interactions, as was recently shown for crown ethers [40]. The size of the pillar[5]arene molecule, with 75 atoms, allows for CSM calculation with the exact algorithm. On the other hand, the flexibility leads to high distortion levels, making the evaluation of the CSM with the approximate permutation-direction iterations approach, quite challenging. Therefore, this system is suitable for benchmark purposes. Although the host itself can be distorted, for the purpose of testing our algorithms we enforced distortion even further and inserted a Li^+^ ion to the center of the host. Previously we showed that the symmetry of the related host, 18-crown-6, is strongly influenced by the presence of alkali ions, particularly Li^+^, and that this distortion is related to the host–guest binding energy [40]. The small size of the ion prevents direct interaction with all the oxygen atoms of the host, forcing the last to fold in order to increase its interaction with the guest. The cavity of pillar[5]arene is larger than 18-crown-6, and it is therefore reasonable to assume that its tendency to distort with respect to either the fivefold or twofold symmetries will be significant when a Li^+^ ion is used as a guest.

#### Conformation analysis

A pillar[5]arene molecule with perfect *D*_5_ symmetry with Li^+^ ion in its center (Fig. 2), was subjected to conformation analysis calculation in the gas phase, using the LowModeMD algorithm [41] implemented in MOE [42]. The Amber10:EHT basis set was used with an energy window of 20 kcal/mol. This calculation created 647 conformers which were filtered to remove duplicates based on their energy, radius of gyration and the CSM with respect to *C*_5_, *C*_2_ and *C*_s_ point groups (were the last serves as chirality measure as explained below). The exact algorithm of the CSM was used for this purpose. We used thresholds of 0.0001 kcal/mol for the energy, 0.05 for the radius of gyration, and a relative threshold of 5% for the CSMs. If the differences between two conformers, for all parameters, were equal or below these thresholds, they were considered equal, and one of them was deleted. The lowest energy conformers within an energy window of 8 kcal/mol were then optimized with Gaussian [43], at the M06/6-31g(d) level including D3-dispersion corrections [44], followed by a second stage of duplicates filtering, as described above. This process resulted with 159 conformers within an energy range of 20.4 kcal/mol. These define our pillar[5]arene data set.

#### Symmetry analysis of pillar[5]arenes

For each conformer of the pillar[5]arene set we calculated the distortion with respect to *C*_5_, *C*_2_ and *C*_s_ point groups for the host molecule without the Li^+^ ion and excluding the hydrogen atoms. Table 1 summarizes results based on the exact CSM algorithm, teaching that none of the conformers in our dataset had perfect *C*_5_ symmetry. Two conformers had perfect *C*_2_ symmetry. We note that S(*C*_s_) is a measure of chirality in this case since the distance of the pillar[5]arene structure from higher order achiral structures (belonging to e.g., *C*_i_, *S*_4_, *S*_6_,… point groups) is larger. As seen in Table 1, all the conformers are chiral to some degree. Although our focus is benchmark of symmetry algorithms, a note about the energy is in place. Generally, direct correlation between energy and symmetry or chirality was not detected for this set. The complex with the minimal energy is not the most symmetric. This is not surprising given the size of the Li^+^ ion compared to the pillar[5]arene cavity and the expected prevalence of host–guest electronic interactions over the symmetry of the host.

**Table 1:** Descriptive statistics for 159 conformers of pillar[5]arene

	Relative Energy (kcal/mol)	*S*(*C*_5_)	*S*(*C*_2_)	*S*(*C*_s_)
Mean	7.4018	9.6912	2.4485	3.5025
Standard deviation	3.6098	1.4267	1.5982	1.1635
Minimum	0.0000	6.5696	0.0000	1.8117
Median	7.1712	9.5697	2.2816	3.3873
Maximum	20.4235	12.9361	7.0320	6.5419

CSM values were calculated with the exact algorithm

**Fig. 3:**
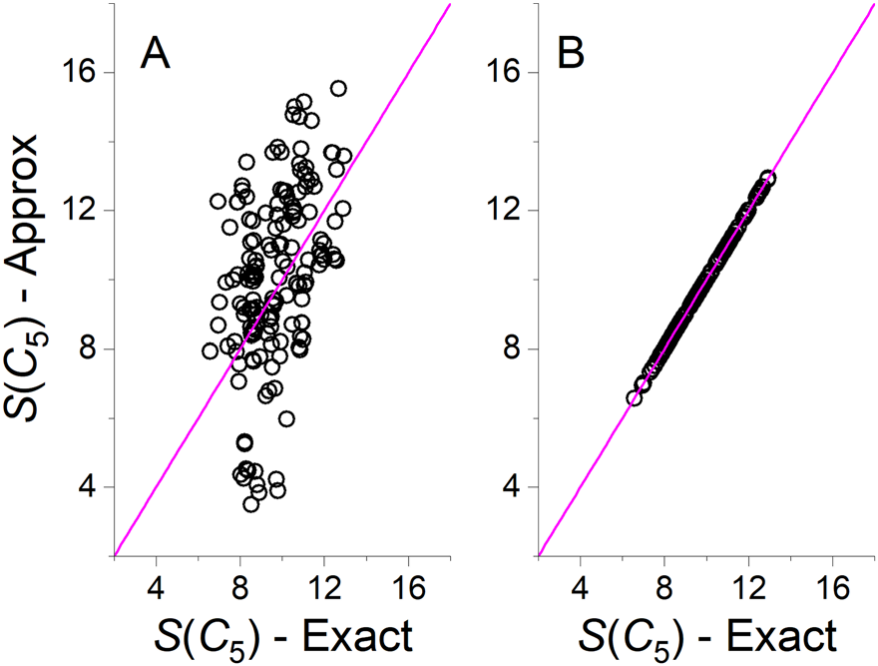
Approximate versus exact values of *S*(*C*_5_) for the pillar[5]arene dataset. **A** Hungarian algorithm **B** Approximate structure preservation algorithm. Magenta line represents the *y* = *x* curve

**Fig. 4:**
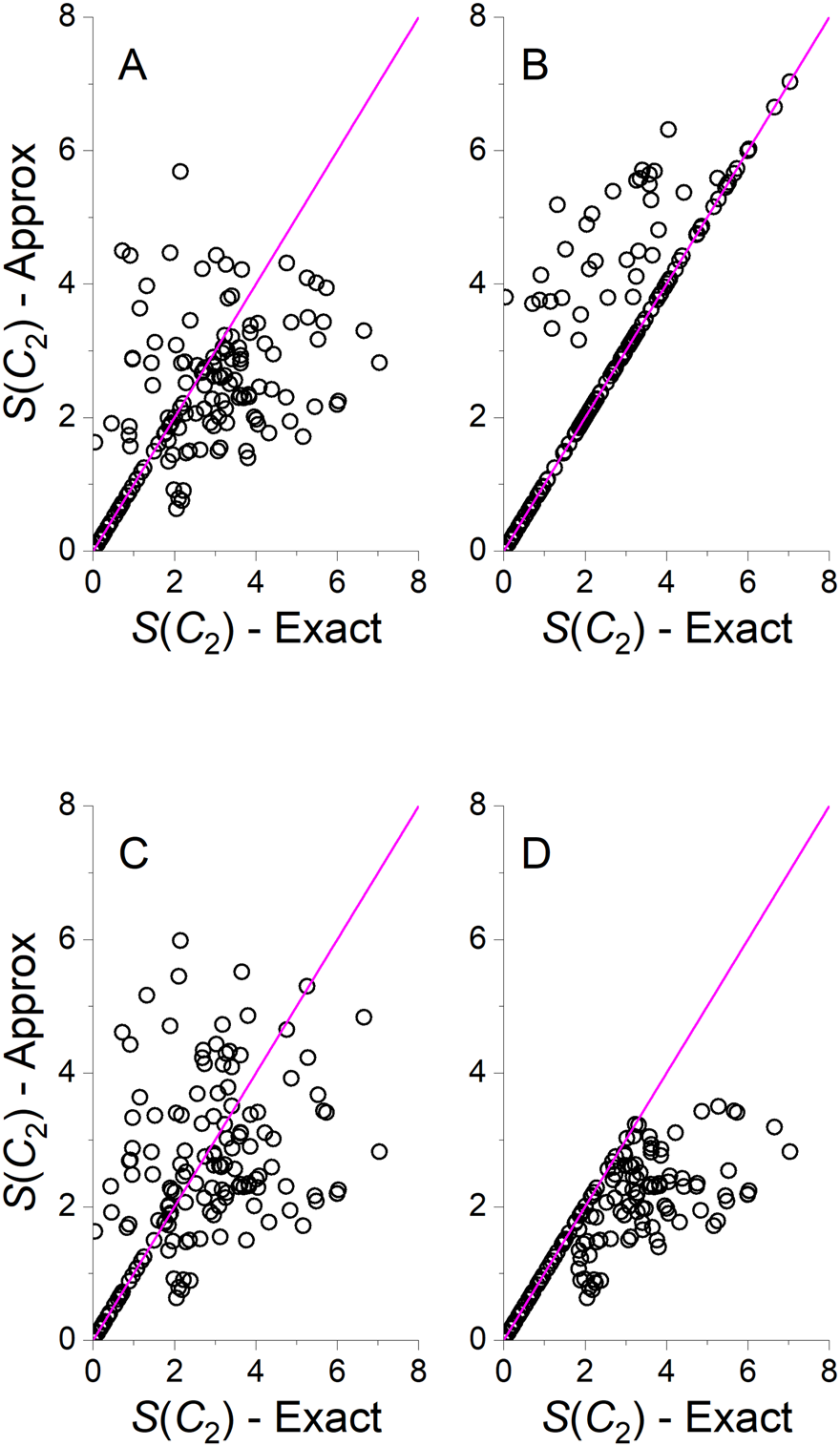
Approximate versus exact values of *S*(*C*_2_) for the pillar[5]arene dataset. **A** Hungarian algorithm **B** Approximate structure preservation algorithm. **C** Greedy algorithm. **D** Fibonacci lattice with 100 directions. Magenta line represents the *y* = *x* curve

Figure 3 presents correlation plots between the exact algorithm and two approximate algorithms: The Hungarian algorithm and the approximate structure-preservation algorithm for *S*(*C*_5_). Figure 4 presents similar correlations for *S*(*C*_2_) with four algorithms (Hungarian, approximate structure preservation, greedy and Fibonacci lattice with 100 directions coupled with the Hungarian algorithm). Additional figures are provided in Additional file 1 : Results for the other approximate approaches for *S*(*C*_5_) which were similar to the Hungarian algorithm (Additional file 1: Fig. S1), and results for *S*(*C*_s_) which were similar to *S*(*C*_2_) (Additional file 1: Fig. S2). It is striking to see how the approximate structure-preserving algorithm outperforms all other algorithms, with perfect correlation for *S*(*C*_5_) and very good correlations for *S*(C_2_) and *S*(*C*_*s*_). For a *D*_5_-symmetric pillar[5]arene, there is a single *C*_5_ rotation axis, and five different *C*_2_ rotation axes. Naturally, when the number of possible permutations is small, it is more likely that an algorithm that is forced to preserve the structure will converge to the correct direction of the rotation axis in space. When the algorithm is not forced to preserve the structure, the calculation can converge with a different permutation, which can either be related to a direction of true rotation axis in the molecule with higher CSM, or a direction that is related to a permutation that does not preserve the structure. Therefore, even a Fibonacci lattice with 100 directions in space provides only an approximate value for the CSM. The Fibonacci method can potentially be improved by increasing the number of directions on the expanse of the computational effort. However, calculations with 500 directions for pillar[5]arene had minor effect on *S*(*C*_5_) and negligible effect on *S*(*C*_2_) and *S*(*C*_s_). When more directions are added, the calculation becomes much slower, and is not justified here. Nevertheless, as discussed above, the method does provide a lower bound to the CSM for *C*_2_ and *C*_s_ symmetries. Comparing the correlations of the Hungarian and greedy algorithms, both of them are less accurate as compared with the other algorithms, with the first showing somewhat better correlation with the exact algorithm.

Another striking finding from Fig. 4 and Additional file 1: Fig. S2 is the high agreement between all the algorithms when the distortion level is low. As seen in Fig. 3B and Additional file 1: Fig. S1, when the CSM is smaller than ~ 2, all the approximate algorithms reach the same CSM as the exact algorithm. A value of 2 for pillar[5]arene means that the distortion is not very high. As an example, Additional file 1: Fig. S3 shows a distorted structure for which *S*(*C*_2_) = 1.8918 superimposed on its nearest *C*_2_-symmetric structure. For other molecules the specific threshold of 2 may vary, but the principle remains: when the distortion is relatively low, all algorithms reach the same CSM. This finding provides us with a very basic guideline for interpreting the values of approximate CSMs: as structural descriptors, they can distinguish between conformers regardless of their symmetry, but their interpretation as structure preserving symmetry measures is most likely guaranteed when the level of distortion is low. When the distortion is high, the nearest symmetric structure may not preserve the chemical essence of the structure in terms of its chemical bonds, yet still maintains its symmetric shape.

Another measure of accuracy of the approximate CSMs is the level of structure preservation with respect to the connectivity map of the molecules [12], displayed in Table 2. The approximate structure preservation algorithm reaches 100% by definition, while the other algorithms preserve 63–90% of the structure, depending on the algorithm and point group. Comparing Tables 1 and 2 we note that structure preservation is better when the CSM is small. Thus, structure preservation with respect to the *C*_2_ and *C*_s_ point groups is better than with respect to *C*_5_. In addition, as one may expect, the structure preservation with the Hungarian algorithm is somewhat better as compared with the greedy algorithm, and further improved upon using the Fibonacci lattice approach, particularly for *C*_2_ and *C*_s_, where more than one direction is possible. Finally we recall that true *C*_s_ symmetry is unlikely for pillar[5]arene and it is used here due to its interpretation as a continuous measure of chirality.

**Table 2:** Structure preservation during approximate CSM calculations for 159 conformers of pillar[5]arene

Algorithm	*S*(*C*_5_) (%)	*S*(*C*_2_) (%)	*S*(*C*_s_) (%)
Structure-preservation	100	100	100
Hungarian	67	86	85
Greedy	63	84	82
Fibonacci lattice (100 directions)	67	90	87
Fibonacci lattice (500 directions)	67	90	87

Last but not least, let us discuss the time of calculation. We ran the code using 1 core on our Intel(R) Xeon(R) Gold 6130 CPU@2.10 GHz server. Table 3 presents the total time for calculating the CSM for 20 conformers (one after the other) taken from the pillar[5]arene data set. Most of the calculations were completed within a few seconds and even less than that, with an average time per molecule of 0.05–6.51 s. The size of the molecule makes the exact algorithm faster than all others, since it scans a smaller number of permutations. Among the approximate approaches, the greedy algorithm is the fastest, with the Hungarian and the approximate structure preservation algorithms coming next with negligible differences between them. The Fibonacci lattice algorithm naturally requires longer time for computation, up to ~ 100 times longer when 500 directions are taken into account.

**Table 3:** User time (in seconds) for consecutive CSM calculation of 20 conformers of Pillar[5]arene

CSM	Exact	Hungarian	Greedy	Structure preservation	Fibonacci with 100 directions	Fibonacci with 500 directions
*S*(*C*_5_)	0.95	2.22	1.53	2.31	18.25	130.16
*S*(*C*_2_)	0.95	1.95	1.14	2.07	14.43	104.40
*S*(*C*_S_)	1.00	1.95	1.20	2.04	16.01	115.72

### C_100_ fullerenes

The Fullerene family of molecules is an allotrope form of carbon, characterized with hollow structures. Fullerene molecules generally display high symmetry, with the most abundant fullerene, C_60_, showing icosahedral symmetry [45]. Chiral fullerenes have also been documented [46, 47]. Fullerenes have numerous applications that exploit their unique symmetry, in host–guest chemistry, solar cells, catalysis, drug design, and cancer treatments [45–50]. Understanding the symmetry and chirality, of fullerenes, particularly when it is only approximate, can shed light on different distortive processes. Here we focus on fullerenes with 100 carbon atoms. Theoretical studies show that this system have 450 topological isomers that follow the isolated-pentagon-rule [51, 52]. C_100_ ions were recently detected experimentally as original constituent of aerosol samples [53].

**Fig. 5:**
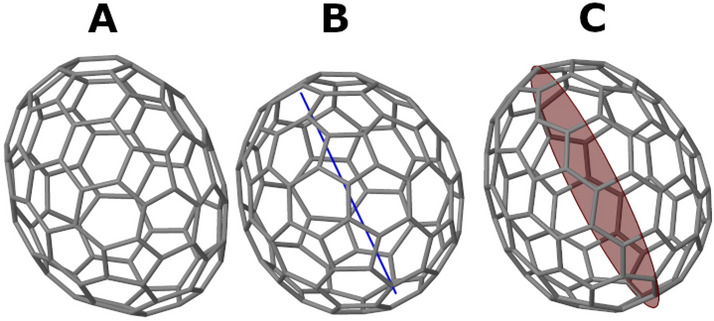
Examples of three isomers of C_100_ from the Yoshida fullerene library that belong to different point groups. **A** Isomer 134 with *C*_1_ symmetry; **B** Isomer 221 with *C*_3_ symmetry shown with blue line; **C** Isomer 126 with *C*_s_ symmetry shown with a red reflection plane

While applicable for an exact CSM calculation, C_100_ fullerene provides an algorithmic challenge for approximate CSM estimation due to variability in topology and the large number of permutations. Coordinates of the 450 isomers of C_100_ fullerene were downloaded from the fullerene library [52] without modifications. These structures are based on the Yoshida’s fullerene library and were further optimized by Tománek using the fast Dreiding-like force field [52, 54]. The data set is divided to 336 topologically asymmetric isomers (that belong to the *C*_1_ point group), 62 isomers with *C*_2_ symmetry, and 31 isomers with *C*_s_ symmetry. The rest 21 isomers display higher symmetry. Examples of three isomers with different symmetries are presented in Fig. 5. Our first goal was to test the set of algorithms on symmetric structures in order to see whether the approximate approaches can identify the correct symmetry. All methods were able to correctly estimate *S*(*C*_2_) for the *C*_2_-symmetric isomers and *S*(*C*_s_) for the *C*_s_-symmetric isomers. The CSM values varied between 0.0000 and 0.0004. The small non-zero CSM values result from negligible numerical inaccuracies of the original coordinates which are reasonable to ignore in this case. No significant differences were found between the different algorithms for these molecules.

The set of 336 asymmetric fullerenes was more challenging. For this set, we calculated the CCM as the minimum of *S*(*C*_s_) and *S*(*C*_i_). The CSM with respect to the *S*_4_ point group was also calculated but found higher than *S*(*C*_s_) for all isomers and will not be discussed here. Since the asymmetry stems from the connectivity map of the molecule and not from conformational distortion, the nearest symmetric or achiral structure, regardless of the algorithm or the point group at hand, can not be a real molecule and atoms' overlap is to be expected for this structure. Nevertheless, the CSM or CCM still have a mathematical meaning of a symmetry or chirality measure, since the reference structure found by the algorithm is the closest structure that belongs to the desired point group, as originally defined by Avnir and coworkers [7, 9, 10].

**Fig. 6:**
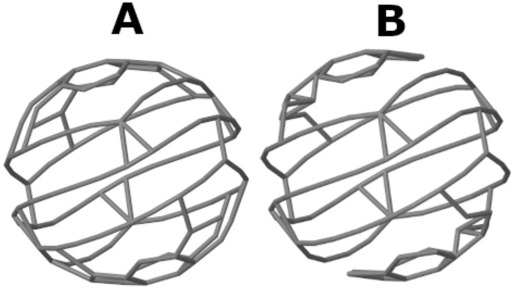
Nearest structure with inversion symmetry for the *C*_1_-isomer #134 of the C_100_ fullerene set calculated by two approximate algorithms. **A** Hungarian algorithm, *S*(*C*_i_) = 0.7296. **B** Greedy algorithm, *S*(*C*_i_) = 1.2325. Original structure is presented in Fig. 5A

To elaborate on this point, let us look at the distortion with respect to inversion symmetry of the asymmetric C_100_ isomers. The exact algorithm returns the value of 100 for *S*(*C*_i_) for each one of the 336 isomers, since the nearest structure with inversion symmetry, that maintains the connectivity map of the molecule, collapses to the center of mass, and all atoms overlap each other. In this sense the exact CSM cannot distinguish between the different isomers. The Hungarian and greedy algorithms on the other hand, find *C*_i_-symmetric structures with lower percentages of atoms' overlap, that retain the hollow shape of the fullerene. Figure 6 displays the nearest *C*_i_-symmetric structures of the *C*_1_ isomer presented in Fig. 5A, calculated with the Hungarian and greedy algorithms. The resulting structures differ from each other and so are the values of *S*(*C*_i_). The ability to distinguish between the original isomers makes the approximate CSMs better structural descriptors for this set of isomers. Therefore, relaxing the requirement of structure preservation is advantageous in this case.

Which of the approximate CSM algorithms provides a more accurate answer to *S*(*C*_i_)? Our main goal is to find the minimal CSM, but we also attempt to reach a reference structure that maintains, as much as possible, the chemical essence of the original molecule. For isomers of C_100_ with *C*_1_ symmetry, calculation of *S*(*C*_i_) with the Hungarian algorithm outperforms the greedy algorithm in both criteria: It finds smaller CSM values (Table 4) and reaches higher percentages of structure preservation as discussed below. The Fibonacci lattice algorithm is unnecessary here since the inversion point must be at the center of mass of the molecule and there is no need to test further directions in space. The approximate structure preserving algorithm reaches the value of 100 as the exact algorithm, with 100% of structure preservation, as expected.

**Table 4:** Descriptive statistics of CSM for 336 asymmetric isomers of C_100_

	CSM	Exact	Hungarian	Greedy	Structure preservation	Fibonacci with 100 directions	Fibonacci with 500 directions
*S*(*C*_i_)	Mean	100	0.8347	1.8239	100		
	Standard deviation	0	0.0843	0.5725	0		
	Minimum	100	0.6138	0.7364	100		
	Median	100	0.8227	1.8086	100		
	Maximum	100	1.1309	3.8252	100		
*S*(*C*_s_)	Mean	26.9289	0.4897	0.7488	26.9289	0.2924	0.2372
	Standard deviation	1.6458	0.1776	0.3218	1.6458	0.1168	0.0939
	Minimum	22.7810	0.0802	0.0802	22.7810	0.0432	0.0426
	Median	26.7276	0.5341	0.8045	26.7276	0.2964	0.2388
	Maximum	31.8030	0.7872	1.6703	31.8030	0.6135	0.5371

**Fig. 7:**
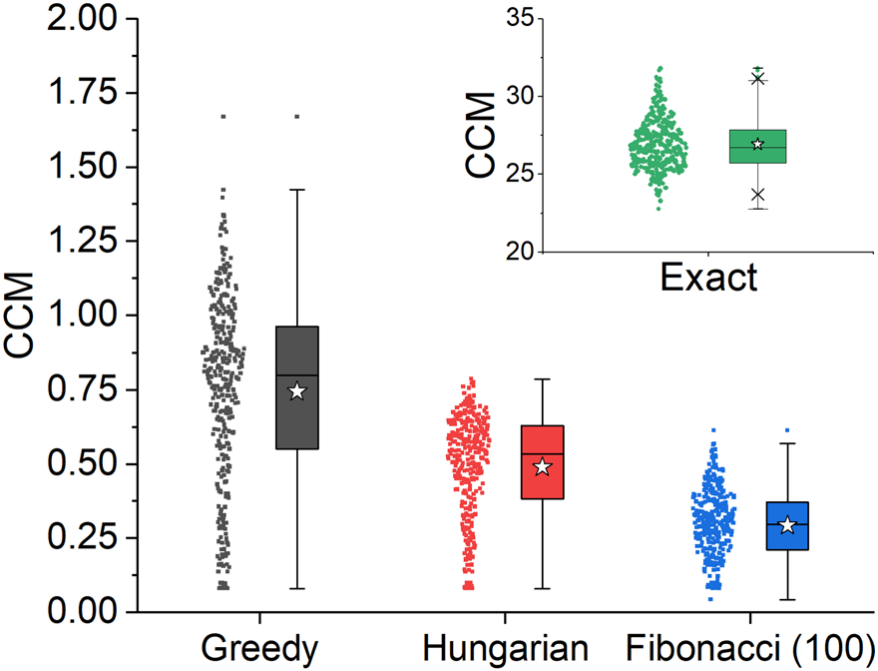
Box and whisker plot of CCM values for 336 asymmetric C_100_ isomers, calculated with different methods. Box boundaries represent 25–75% of the data, horizontal line within the box is the median and the white star is the mean value in each box

**Fig. 8:**
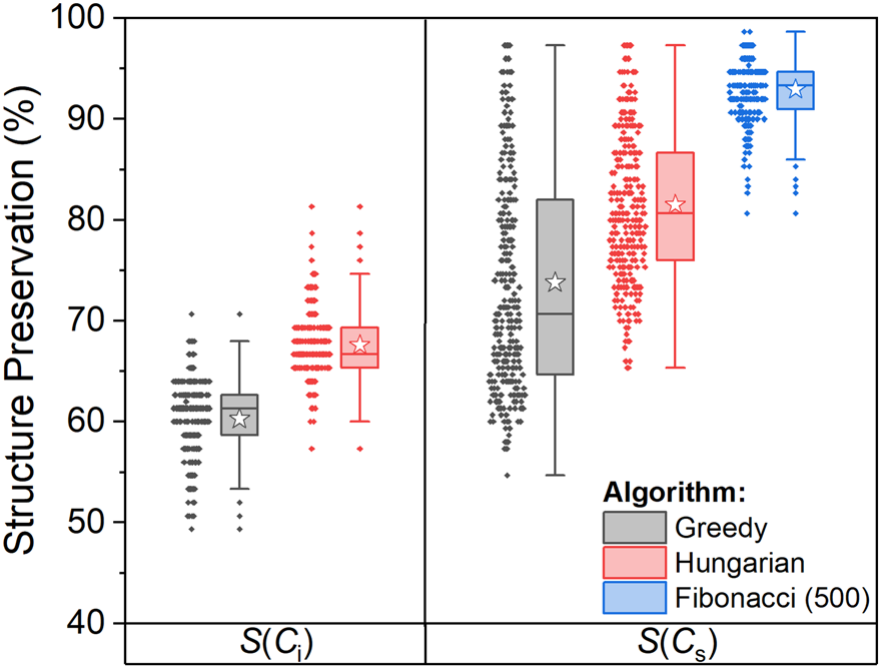
Box and whisker plot of the percentages of structure preservation for approximate CSM calculations for 336 asymmetric C_100_ isomers. Left: *S*(*C*_i_). Right: *S*(*C*_s_). Box boundaries represent 25–75% of the data, horizontal line within the box is the median and the white star is the mean value in each box

Repeating this analysis for *S*(*C*_s_) we found smaller CSM values as compared with *S*(*C*_i_), for all algorithms and all the isomers. We can thus claim that CCM = *S*(*C*_s_) in this case, that is, the chirality of the C_100_ isomers stems from the lack of reflection symmetry. Figure 7 presents a box and whisker plot of the CCM calculated with the different algorithms. Using the exact algorithm (as well as the approximate structure preserving algorithm) the nearest structure with reflection symmetry collapses to a planar surface, with a mean CSM of 27. In other words, the exact CSM can be interpreted as a planarity measure in this case. With the approximate algorithms, the nearest symmetric structures maintain the ellipsoid shape by overlapping fewer atoms, leading to much smaller CSM values. We note that unlike the exact algorithm, the approximate algorithms predict that different isomers will have different chirality levels. Additional file 1: Fig. S4 displays the nearest symmetric structure with reflection symmetry that was obtained for isomer #134 presented in Fig. 5A. In this sense the approximate CSM is a good 3D-shape descriptor. The Fibonacci lattice algorithm improves the Hungarian algorithm and finds reference structures with smaller CSM values. Increasing the number of directions to 500 improves the results even further, but with minor effect, therefore not shown here. Figure 8 shows that as compared with *S*(*C*_i_), structure preservation for reflection symmetry is better with all algorithms, and further improves with the Fibonacci lattice algorithm. Table 4 summarizes descriptive statistics of these calculations and Table 5 presents data on the time of calculation for a subset of 20 C_100_ fullerenes on our server. Unlike the pillar[5]arene data set, here the exact algorithm was slower than some of the approximate algorithms. Particularly for S(*C*_i_), an approximate algorithm that does not attempt to preserve the structure is much faster than an exact calculation. The greedy algorithm was the fastest algorithm for both S(*C*_i_) and S(*C*_s_).

**Table 5:** User time (in seconds) for consecutive CSM calculation of 20 isomers of C_100_ fullerenes

CSM	Exact	Hungarian	Greedy	Structure preservation	Fibonacci with 100 directions	Fibonacci with 500 directions
*S*(*C*_i_)	15.71	1.61	1.10	91.84		
*S*(*C*_s_)	15.54	14.15	2.09	460.65	171.72	857.00

### Metal organic frameworks

**Fig. 9:**
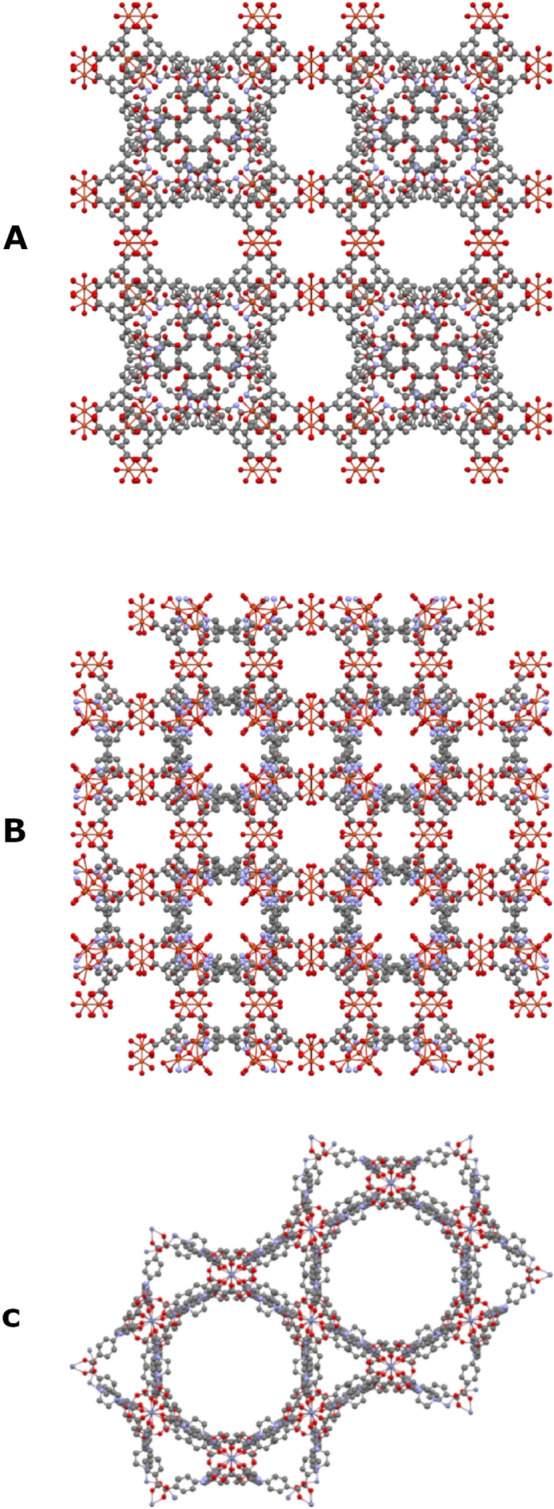
A set of three MOFs, viewed along their unit cell c axis, without the hydrogens atoms taken from the COD IDs: **A** 4002650, **B** 4003149, **C** 4002646

As a third challenge we focused on crystal structures for which the exact algorithm cannot scan the permutation space in a reasonable time frame. Our purpose was to test whether perfect symmetry can be identified by approximate CSM algorithms, to estimate the time of calculation, and to evaluate the extent by which the structures of the initial sets of coordinates are preserved in the final sets. Coordinates of three crystals of MOFs with very large unit cells were retrieved from the Crystallographic Open Database (COD) [55]. Structures that contain all the molecules whose centroids fit inside a single unit cell were constructed using Mercury [56], and are presented in Fig. 9. Continuous symmetry measures with respect to several point groups were calculated with the Hungarian algorithm and are summarized in Table 6. Perfect symmetry, as expected from the space group, is clearly identified with 100% structure preservation. Our method correctly predicts that the unit cell of UHM-25-Ala-Boc [57] (Fig. 9A) that belongs to the P432 space group is chiral, while the other two structures are achiral. We note that the non-zero CSM values of UHM-25-Ala-Boc are relatively small. This stems from the large number of atoms that significantly increases the normalization factor, $D$ in (1). Results for the greedy algorithm were similar to the Hungarian algorithm, with a bit higher CSM in the lack of perfect symmetry in most of the cases (Additional file 1: Table S1), on the expense of lower structure preservation (Additional file 1: Table S2). With regards to time of calculation, the greedy algorithm was significantly faster, and ranged between ca. 4–35 s per structure. The Hungarian algorithm required longer time in most cases, ranging between ca. 4–221 s per structure (Additional file 1: Table S3). The Fibonacci lattice algorithm required much longer calculation time and is not justified here.

**Table 6:** Approximate CSMs of unit cells of MOFs, calculated with the Hungarian algorithm

Name	UHM-25-Ala-Boc [57]	rht-MOF-pyr [58]	MUF-22 [59]
COD ID	4002650	4003149	4002646
Formula	C_36_H_32_Cu_2_NO_13_	C_33_H_15_Cu_6_N_6_O_19_	C_48_H_36_N_4_O_12_Zn_3_
Space group	P432	${\text{Fm}}\overline{3}{\text{m}}$	${\text{R}}\overline{3}{\text{c}}$
Number of atoms	12,000	7552	2565
*S*(*C*_s_)	0.0601	0.0001	12.7755
*S*(*C*_i_)	0.0608	0.0000	0.0000
*S*(*C*_2_)	0.0000	0.0001	12.7755
*S*(*C*_3_)	0.0000	0.0000	34.0055
*S*(*C*_4_)	0.0000	0.0000	35.6589
*S*(S_4_)	0.0608	0.0000	35.6611
*S*(S_6_)	0.0608	0.0000	40.8096

### Guidelines for effective usage

The CSM code offers several algorithms and many parameters that affect the accuracy and speed of the calculation, as well as the chemical and mathematical interpretation of the results. Choosing between all possible options is not always trivial, and may benefit from a trial and error strategy. Nevertheless, based on our current findings and previous studies [12, 13], we provide here several guidelines for the choice of algorithms for CSM and CCM analysis of molecular structures.

Small-to-medium sized molecules are suitable for an exact calculation, based on the structure preserving permutation algorithm [12]. The number of atoms is the first indicator of size. A molecule with up to several hundreds of atoms is generally considered medium sized here, but the bonding structure and the size of the symmetry equivalent atoms' groups is also important, as they affect the number of possible permutations. Ignoring the hydrogen atoms is a common workaround that reduces the number of permutations and can speed up the calculation. While the hydrogen atoms naturally affect the CSM value of a given molecule, they often have minor effect on the overall distortion trends of a set of related molecules. Another option for size reduction is to analyze the core structure of large molecules, particularly when this structure has a symmetric topology. Sets of molecular derivatives with a common skeleton, can especially benefit from this type of analysis [15].

Large structures for which the exact algorithm is not practical, are handled with approximate CSM calculations. Several algorithms can be used, and are selected as an interplay between calculation time and accuracy:

1. The Hungarian algorithm is our default choice for approximate CSM calculation, presenting a reasonable compromise between speed and accuracy. This algorithm also excels in distinguishing between different molecules, and as such can be used with medium-sized molecules as well, like the fullerenes discussed above.
2. The approximate structure-preserving algorithm is more accurate than the Hungarian algorithm in its ability to find permutations that preserve the chemical essence of the structure. The resulting CSM values correlate well with the corresponding values of the exact algorithm, as seen for the sets of pillar[5]arene complexes and the fullerenes presented here. The drawback is that these calculations take longer time.
3. The greedy algorithm is often the fastest choice among all approximate algorithms. It is a relatively crude approximation, showing the lowest accuracy in terms of structure preservation. Nevertheless, like the Hungarian algorithm it excels in terms of distinguishing between molecules.
4. The Fibonacci sphere algorithm with up to 100 directions can improve the accuracy of the Hungarian algorithm and find permutations that lead to smaller CSM values. The drawback is a longer calculation time. It is particularly useful for molecules with large equivalence groups of atoms, (e.g., highly symmetric nanoparticles) for which a slight change of the initial direction can help the code to find a better permutation. Increasing the number of directions beyond 100 can increase the accuracy along with the time of calculation.
5. In the special case of protein oligomers, one first need to make sure that chains' length and sequence are equalized. Then the Hungarian algorithm is recommended together with special features of sequence preservation discussed in our previous publication [13].

Calculation of the CCM follows a similar strategy as the CSM with one additional guideline. In many cases of chiral or asymmetric topology, the chirality stems from the lack of reflection symmetry. That is, *S*(*C*_s_) is expected to be smaller than *S*(*C*_i_) or *S*(*S*_n_) with n > 2. Therefore, it is often enough to calculate *S*(*C*_s_) in order to speed up the calculation of the CCM, as was done here for pillar[5]arenes.

### The CSM and the alignment problem

The problem of calculating the continuous symmetry measure is somewhat related to the problem of molecular alignment and RMSD (root mean square deviation) calculations, where one is interested in finding the best fit between two structures.

More concretely, given two sets of atom positions—${{\mathbf{P}}} = \left\{ {{{\mathbf{P}}}_k :\,\,1 \le k \le N} \right\}$ and ${\mathbf{Q}} = \left\{{\mathbf{Q}}_k :\,\,1 \le k \le N \right\}$, alignment algorithms look for a permutation $\pi$ of $\left\{ {1,2,\ldots,N} \right\}$ and a spatial transformation $T$ such that the RMSD is minimized:

8 $$RMSD = \sqrt {{\frac{1}{N}\sum_{k = 1}^N {\left| {TP_k - Q_{\pi (k)} } \right|^2 } }}$$

This problem has been widely discussed in the literature [60–62].

If we take ${{\mathbf{P}}} = {{\mathbf{Q}}}$, then the alignment problem becomes very similar to the problem of finding the CSM. However, when we look for symmetry, we have to exclude the trivial solution where $T$ is the identity transformation. We also impose restrictions on the operation $T$. In the context of point symmetry, $T$ must generate a *finite* group. This means, for example, that if $T$ is a rotation around an axis vector, then the angle of rotation must be a multiple of $360^{\circ} /n$ where $n$ is the order of the symmetry operation. In contrast, when we look for mere alignment, $T$ can be a rotation at any angle.

The continuous shape measure, CShM, originally developed by Pinsky and Avnir [11] as an extension of the CSM method, follows a similar strategy. It becomes a symmetry measure when the reference shape is symmetric. Various applications of this approach for small molecules were published through the years for the analysis of e.g., coordination complexes and their distortion pathways, particularly with respect to platonic solids [30, 31, 63]. The techniques described here could be applied, with some adjustments, to calculate shape measures as well as solving the alignment problems for large molecules. Progress in this direction has already been documented, in the form of alignment algorithms that calculate the RMSD while taking symmetry into considerations, utilizing, for example, the Hungarian algorithm [64, 65] and the greedy algorithm with some level of structure preservation [66].

## Conclusions

The continuous symmetry and chirality measures estimate the distance between a given molecule and its nearest symmetric (or achiral) structure. Preserving the connectivity map of the original molecule, although desired, was not a requirement in the original CSM algorithm [7, 10]. Consequently, the approach was limited by the size of molecules that could be handled, an obstacle that was partially overcame by applying an approximate approach based on a permutation-direction search using the greedy algorithm [9]. In recent years, our group considerably improved the method. We introduced the concept of structure preserving permutations in order to reduce the number of permutations the code needs to scan, while preserving the connectivity map of the original molecule [12]. We further improved the approximate algorithm for protein homomers by applying the Hungarian algorithm instead of the greedy algorithm in conjunction with utilizing the sequence structure to reduce the number of permutations [13]. In this work, we took the method one step forward and developed a set of approximate algorithms that are suitable to *any type* of molecular structure. Our main goal is to provide a quantitative estimation to the symmetry and chirality content of molecules of any size. Our set of approximate CSM algorithms should thus be viewed as a collection of 3D geometrical descriptors for global structural analysis that fulfills the main criteria for good descriptors, in terms of interpretability, ability to differentiate between isomers and conformers, applicability for local structures, continuity, usage simplicity and efficiency [67]. As such, these descriptors are suitable for characterizing structural changes and play part in QSAR/QSPR modeling.

The accuracy of all the approximate methods described in this paper depends on the distortion level of the molecule with respect to the desired symmetry point group *G*. For molecules which are only slightly distorted, all the algorithms tend to find a permutation that preserve the structure (in terms of its connectivity map) either fully or with a high preservation rate. For highly distorted molecules, this does not always hold, as permutations which do not preserve the structure could attain lower values of the target function, thereby creating a bias between the exact and approximate CSMs.

Based on our calculations of three different sets of molecules (Pillar[5]arenes complexes, C_100_ fullerenes, and MOFs), guidelines for effective usage of the different algorithms were specified. These are based on the ability of the code to find structure preserving permutations (wherever relevant), the speed of the calculation and the ability of the CSM to distinguish between the studied molecules. In summary, we emphasize the ease and efficiency of using the CSM and CCM approach for structural analysis, which make them applicable as robust three-dimensional geometrical descriptors that can be used to follow dynamical processes and statistical studies on quantitative structure–activity and structure-properties relationships.

### Supplementary Information

**Additional file 1.** Additional Tables and Figures that support the research findings.

## Data Availability

The datasets supporting the conclusions of this article are available at: https://continuous-symmetry.github.io/CSM-OUI/Data. Free online calculators of the CSM and CCM for small molecules and protein homomers are available at https://csm.ouproj.org.il. The CSM software is freely available. Project name: CSM; Project homepage: https://github.com/continuous-symmetry-measure/csm; Archived version: 1.3.7.b1; Operating system(s): Linux, Windows; Programming language: Python, c++; Other requirements: OpenBabel 3.1.1, Cython 0.29.14, scipy 1.7.3, c++ compiler, conda, numpy; License: GNU-GPL version 2; Any restrictions to use by non-academics: Not applicable.

## Abbreviations

CCM: Continuous chirality measure
COD: Crystallographic open database
CShM: Continuous shape measure
CSM: Continuous symmetry measure
MOF: Metal organic framework
QSAR: Quantitative structure activity relationship
QSPR: Quantitative structure property relationship
RMSD: Root mean square deviation

## Footnotes

1 The concise presentation of the Fibonacci lattice in Eqs. (5) and (6) is based on a code that was published under the Stack Overflow platform by the user Fnord. After testing this code and confirming its reliability, we applied it in the CSM software. The original code is available at: https://stackoverflow.com/questions/9600801/evenly-distributing-n-points-on-a-sphere. Accessed 2023, July 28.
