# Supplementary material for: Continuous symmetry and chirality measures: approximate algorithms for large molecular structures
Source: J Cheminform. 2023 Nov 9;15:106. doi: 10.1186/s13321-023-00777-x (PMC10636902; doi:10.1186/s13321-023-00777-x)
Supplement: Supplementary file 1 — Additional file 1. Additional Tables and Figures that support the research findings. [file 13321_2023_777_MOESM1_ESM.pdf]

Additional File 1

Continuous Symmetry and Chirality Measures: Approximate Algorithms for Large Molecular Structures

Gil Alon[1*], Yuval Ben-Haim[2] and Inbal Tuvi-Arad[2*]

[1] Department of Mathematics and Computer Science, The Open University of Israel, Raanana, Israel.

[2] Department of Natural Sciences, The Open University of Israel, Raanana, Israel.

Contents

Coordinates....2
Figure S1....2
Figure S2....3
Figure S3....4
Table S1....4
Table S2....5
Table S3....5

Coordinates of the datasets discussed in the paper:

- Pillar[5]arenes and metal organic frameworks: https://continuous-symmetry.github.io/CSM-OUI/Data/
- C_{100} fullerenes: https://nanotube.msu.edu/fullerene/fullerene-isomers.html

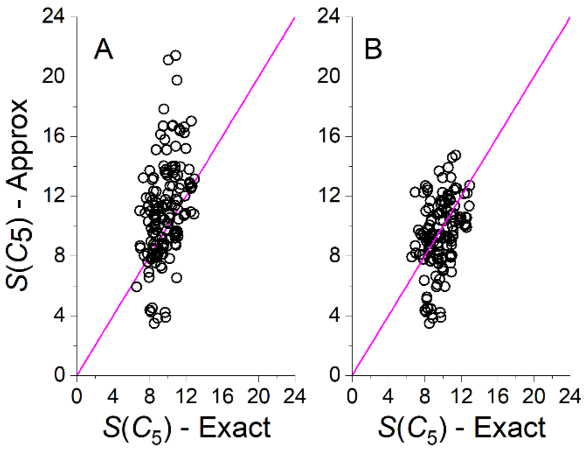

Figure S1. Approximate versus exact values of $S(C_5)$ for the pillar[5]arene dataset. A. Greedy algorithm B. Fibonacci Lattice algorithm with 100 directions. Magenta line represents the $y = x$ curve.

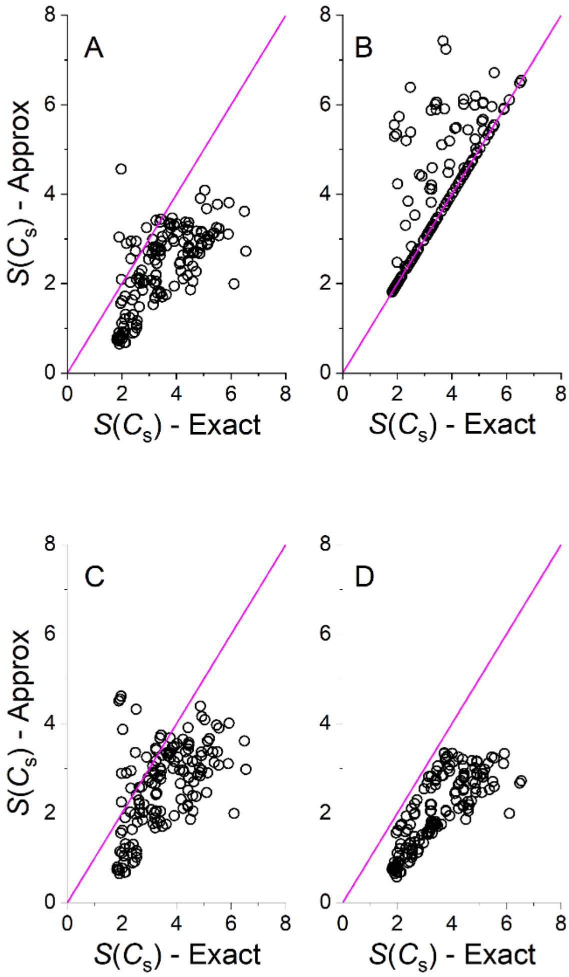

Figure S2. Approximate versus exact values of $S(C_s)$ for the pillar[5]arene dataset. **A.** Hungarian algorithm **B.** Approximate structure preservation algorithm. **C.** Greedy algorithm. **D.** Fibonacci sphere with 100 directions. Magenta line represents the $y = x$ curve.

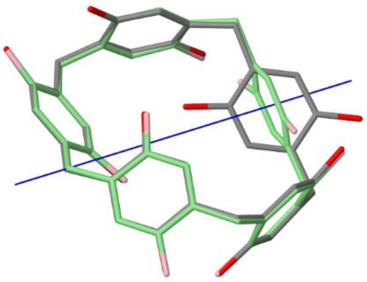

Figure S3. Superposition of Pillar[5]arene with approximate C_2 symmetry (grey-red) and its C_2-symmetric counterpart as found by the CSM code with the exact algorithm (green-pink). The blue line represents the C_2 axis of the symmetric structure. $S(C_2)$ = 1.8918. The CSM is relatively small since the distortion stems from conformational change of a single ring.

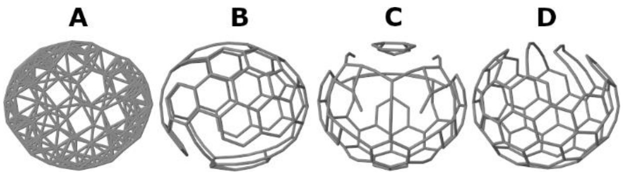

Figure S4. Nearest structure with reflection symmetry for the C_1-isomer #134 of the C_{100} fullerene set calculated by different approximation algorithms. **A.** Exact algorithm, $S(C_s)$=26.9511; **B.** Hungarian algorithm, $S(C_s)$=0.3901. **C.** Greedy algorithm, $S(C_s)$=0.8084; **D.** Fibonacci lattice algorithm with 100 directions, $S(C_s)$=0.3378. Original structure is presented in Figure 5A.

Table S1. Metal organic frameworks: CSMs of unit cells calculated with the greedy algorithm.

**Name**	UHM-25-Ala-Boc[51]	rht-MOF-pyr[52]	MUF-22[53]
**COD[49] ID**	4002650	4003149	4002646
**Formula**	$C_{36}H_{32}Cu_2NO_{13}$	$C_{33}H_{15}Cu_6N_6O_{19}$	$C_{48}H_{36}N_4O_{12}Zn_3$
**Space group**	P432	Fm$\bar{3}$m	R$\bar{3}$c
**Number of Atoms**	12,000	7552	2565
$\boldsymbol{S(C_s)}$	0.0717	0.0000	12.8082
$\boldsymbol{S(C_i)}$	0.0717	0.0000	0.0000
$\boldsymbol{S(C_2)}$	0.0000	0.0000	12.8082
$\boldsymbol{S(C_3)}$	0.0000	0.0000	39.6989
$\boldsymbol{S(C_4)}$	0.0000	0.0000	49.2383
$\boldsymbol{S(S_4)}$	0.0717	0.0000	36.8784
$\boldsymbol{S(S_6)}$	0.0717	0.0000	40.8964

Table S2. Metal organic frameworks: Structure preservation for CSM calculations of single unit cells.

**Algorithm**	Hungarian	Hungarian	Hungarian	Greedy	Greedy	Greedy
**COD[49] ID**	4002650	4003149	4002646	4002650	4003149	4002646
**Number of Atoms**	12,000	7552	2565	12,000	7552	2565
$\boldsymbol{S(C_s)}$	93.73%	100.00%	97.07%	91.59%	100.00%	97.84%
$\boldsymbol{S(C_i)}$	93.53%	100.00%	100.00%	91.59%	100.00%	100.00%
$\boldsymbol{S(C_2)}$	100.00%	100.00%	97.07%	100.00%	100.00%	97.84%
$\boldsymbol{S(C_3)}$	100.00%	100.00%	93.03%	100.00%	100.00%	73.80%
$\boldsymbol{S(C_4)}$	100.00%	100.00%	92.06%	100.00%	100.00%	82.96%
$\boldsymbol{S(S_4)}$	93.53%	100.00%	92.20%	91.59%	100.00%	85.02%
$\boldsymbol{S(S_6)}$	93.53%	100.00%	92.47%	91.59%	100.00%	88.26%

Table S3. Metal organic frameworks: User time (in seconds) for CSM calculations of single unit cells.

**Algorithm**	Hungarian	Hungarian	Hungarian	Greedy	Greedy	Greedy
**COD[49] ID**	4002650	4003149	4002646	4002650	4003149	4002646
**Number of Atoms**	12,000	7552	2565	12,000	7552	2565
$\boldsymbol{S(C_s)}$	184.89	57.46	20.27	22.6	13.25	7.79
$\boldsymbol{S(C_i)}$	12.21	9.06	3.88	7.66	5.59	3.78
$\boldsymbol{S(C_2)}$	40.22	57.78	19.93	13.17	12.76	7.9
$\boldsymbol{S(C_3)}$	137.55	20.46	10.94	20.07	7.45	5.73
$\boldsymbol{S(C_4)}$	27.22	136.81	8.53	10.14	16.24	6.24
$\boldsymbol{S(S_4)}$	220.92	136.06	8.49	34.88	10.37	5.84
$\boldsymbol{S(S_6)}$	145.49	21.38	11.79	27.96	7.74	9.45
